# Polarization modulation with optical lock-in detection reveals universal fluorescence anisotropy of subcellular structures in live cells

**DOI:** 10.1038/s41377-021-00689-1

**Published:** 2022-01-01

**Authors:** Meiling Guan, Miaoyan Wang, Karl Zhanghao, Xu Zhang, Meiqi Li, Wenhui Liu, Jing Niu, Xusan Yang, Long Chen, Zhenli Jing, Micheal Q. Zhang, Dayong Jin, Peng Xi, Juntao Gao

**Affiliations:** 1 Department of Biomedical Engineering, College of Future Technology, Peking University, Beijing, 100871 China; 2 UTS-SUStech Joint Research Centre for Biomedical Materials & Devices, Department of Biomedical Engineering, College of Engineering, Southern University of Science and Technology, Shenzhen, Guangdong China; 3 MOE Key Laboratory of Bioinformatics, Bioinformatics Division, Center for Synthetic & Systems Biology, BNRist, Beijing, China; 4 Center for Synthetic & Systems Biology; Department of Automation, Tsinghua University, Beijing, 100084 China; 5 Beijing Institute of Collaborative Innovation, Beijing, 100094 China; 6 Department of Biological Sciences and Center for System Biology, The University of Texas at Dallas, Richardson, 75080 USA; 7 School of Medical Sciences, Tsinghua University, Beijing, 100084 China; 8 Institute for Biomedical Materials and Devices (IBMD), Faculty of Science, University of Technology Sydney, Sydney, NSW 2007 Australia; 9 National Biomedical Imaging Center, Peking University, Beijing, 100871 China

**Keywords:** Super-resolution microscopy, Polarization microscopy

## Abstract

The orientation of fluorophores can reveal crucial information about the structure and dynamics of their associated subcellular organelles. Despite significant progress in super-resolution, fluorescence polarization microscopy remains limited to unique samples with relatively strong polarization modulation and not applicable to the weak polarization signals in samples due to the excessive background noise. Here we apply optical lock-in detection to amplify the weak polarization modulation with super-resolution. This novel technique, termed optical lock-in detection super-resolution dipole orientation mapping (OLID-SDOM), could achieve a maximum of 100 frames per second and rapid extraction of 2D orientation, and distinguish distance up to 50 nm, making it suitable for monitoring structural dynamics concerning orientation changes in vivo. OLID-SDOM was employed to explore the universal anisotropy of a large variety of GFP-tagged subcellular organelles, including mitochondria, lysosome, Golgi, endosome, etc. We found that OUF (Orientation Uniformity Factor) of OLID-SDOM can be specific for different subcellular organelles, indicating that the anisotropy was related to the function of the organelles, and OUF can potentially be an indicator to distinguish normal and abnormal cells (even cancer cells). Furthermore, dual-color super-resolution OLID-SDOM imaging of lysosomes and actins demonstrates its potential in studying dynamic molecular interactions. The subtle anisotropy changes of expanding and shrinking dendritic spines in live neurons were observed with real-time OLID-SDOM. Revealing previously unobservable fluorescence anisotropy in various samples and indicating their underlying dynamic molecular structural changes, OLID-SDOM expands the toolkit for live cell research.

## Introduction

Due to the diffraction limit, the spatial resolution of conventional wide-field fluorescence microscopy is about 200 nm, limiting its application in studying the fine structural changes, interactions, and function of subcellular. Over the past decades, super-resolution fluorescent microscopy brought the vision of the structure and interaction of the subcellular world beyond the diffraction limit. In super-resolution microscopy, a route fully exploited is to introduce a different dimension to modulate the fluorescent intensity^1^. For example, (f)PALM/STORM^2–4^ breaks the resolution barrier by ON-OFF modulating or separating the molecules in the time domain. STED^5,6^ shrinks the point spread function (PSF) by filtering out the stimulated emission within the donut area with spectral-domain modulation. For computational imaging, SIM expands the high-frequency space with structured illumination, resulting in a twofold increase in resolution relative to that of the wide field. For the sake of further improving the imaging performance and speed, deconvolution has been fully applied to a variety of computational imaging and the post-processing of super-resolution imaging, such as 3D fluorescence imaging^7^, confocal^8^, 3D SIM^9^, SOFI^10^, SMLM^11^, and STED^12^, providing a more powerful tool for dense samples imaging in the condition of weak fluorescence intensity.

The dipole orientation of a fluorescent molecule is an inherent physical nature in the fluorescent excitation/emission process, which can be easily modulated and detected. Orientation measurement has found widespread use in studying biological mechanisms associated with molecular arrangement and rotation. Fluorescence polarization microscopy (FPM) is a classical microscopic technique to measure the orientation of bio-macromolecules through polarization^13^. It is first applied in studying lipid membrane^14^, in which the orientation disorder in cell membranes has been found due to cholesterol depletion^15^ or cytoskeleton perturbation^16^. Furthermore, FPM imaging could monitor the dynamic protein activation during the molecular process, such as calcium flow or protein interaction^17^, and measure the orientation of the cytoskeleton, such as actin^18,19^, microtubule^20^, or septin^21^. Besides, orientation measurement has been demonstrated in monitoring molecular transformations, such as nuclear pore complex^22^, myosin^23,24^, kinesin^25^, ATPase^26^, etc. Introducing polarization modulation into super-resolution imaging has achieved tremendous progress recently^27–36^. Examples of this approach include single-molecule position and orientation imaging^28,29,36^, super-resolution dipole orientation mapping (SDOM)^32,34^, and polarized structured illumination microscopy (pSIM)^35^.

However, super-resolution polarization microscopy and orientation measurement have been applied only to a handful of samples discussed above with apparent fluorescence anisotropy. The fluorescence anisotropy is decreased due to various reasons: (1) rotational diffusion of the fluorophores; (2) wobbling of the label linker; (3) homogeneous distribution and flexibility of the target biomolecules. In samples with low fluorescence anisotropy, the read-out signal is usually buried under the system noise, which appears to be polarization invariant. Biologists often hesitate on the analysis of fluorescent dipole orientation, because they could hardly observe it at low signal-to-noise level. Whereas, fluorescence anisotropy does exist universally in biological structure due to its heterogeneity in nature. Uncovering the heterogeneity would contribute to a better understanding of many unresolved biological questions and mechanisms with their structure and orientation/position alterations.

In this work, we developed a comprehensive method termed optical lock-in detection super-resolution dipole orientation mapping (OLID-SDOM) for weak fluorescence anisotropy mapping in universal subcellular ultrastructure. A frequency-domain optical lock-in detection (OLID) was employed to extract the dipole signal, which was modulated by polarized excitation at a given frequency. Super-resolution reconstruction was then applied to reduce spatial averaging of the polarized signal, which would also increase the fluorescence anisotropy. In contrast to ON-OFF modulation-based OLID, polarization modulation-based OLID applies to most organic dyes and fluorescent proteins, especially for the most common green fluorescent protein (GFP) which could be easily labeled to proteins for live-cell imaging. In this work, fluorescence anisotropy was first observed in various subcellular organelles (e.g., lysosomes, endosomes, Golgi, etc.) in live yeast cells and live mammalian cells. Next, we investigated the dynamic orientation alteration of the spine head during outreaching, which only happens on the mono-budding spine. These results on live subcellular organelles and live neuron spines indicate that OLID-SDOM holds great potential for a wide range of biological applications, particularly in which the dipole nature is neglected previously.

## Results

### OLID via polarization modulation

In FPM, the background noise can easily overwhelm the weak polarized fluorescence signal. Background noise could come from various sources, such as the scattered fluorescence, auto-fluorescence, Poisson noise, etc. Similar to lock-in amplification widely applied in electronics, OLID has been demonstrated to be a powerful tool for contrast-enhanced imaging^37,38^. OLID utilizes the photoswitchable property of fluorescent probes, including fluorescent dyes, proteins, and inorganic probes^39–42^. The fluorescent probes exhibit a non-linear response to the optical modulation, most of which behave switchable between fluorescent and dark states. Typically, two lasers with different wavelengths are used to switch the fluorescent probes ON or OFF for a few cycles, requiring synchronized control. Since the background noise remains unmodulated or behaves linear response, lock-in detection can extract the modulated signal same as modulation frequency, and also detect its phase. The lock-in detection method based on cross-correlation calculation requires both the signal of interest and the reference. The former exists in the time curve of each pixel of the acquired images, and the latter could be obtained either from the modulating hardware or from the acquired images. ON-OFF modulation-based OLID methods require specifically designed probes and controlling of different pulsed laser sources. In addition, hardware synchronization or image analysis method is necessary for the generation of the reference signal.

**Fig. 1:**
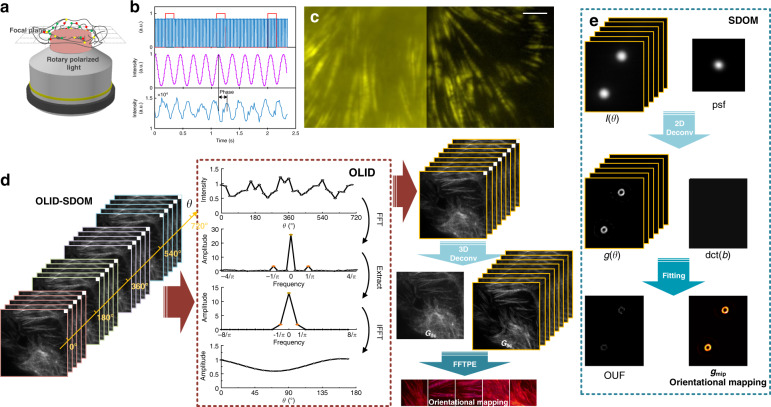
Principle of OLID. **a** Rotary-linear-polarized illumination scheme of OLID. **b** Top: the sync signals of camera readout (blue line) and motor rotation pulse (red line). Middle: intensity curve of the laser after passing through a polarizer parallel to the *X*-axis of the system on the focal plane. Bottom: the emission intensity curve of a GFP molecule on the focal plane under polarization modulated excitation. There are four modulation cycles in one motor rotation cycle. **c** Alexa Flour 561 labeled F-actin in fixed mouse kidney sections, with wide-field image on the left, OLID image on the right. Scale bar: 5 μm. **d** The flowchart of OLID-SDOM. The data of four modulation cycles at each pixel were transformed by 1D Fourier transform, and then we extracted the modulated frequency component and generated a 1D single period data. After processing each pixel, 3D deconvolution of the obtained single period image, named as OLID image, was carried out to obtain the *G*_dc_(*R*), and *G*_ac_(*R,θ*), and orientation mapping was extracted by FFT phase extraction. **e** The flowchart of SDOM. 2D sparse deconvolution was performed for super-resolution reconstruction and the least-squared fitting was performed for orientation mapping

Here we proposed a simplified OLID system based on polarization modulation of the dipole excitation or linear dichroism. Polarization modulation is achieved with a rotary, linear polarized light that irradiated the sample at different polarization angles (Fig. 1a) by a rotatory half-wave plate (HWP). The hardware was identical to a linear dichroism system, in which the fluorescence signal excited at specific polarization angle was simultaneously recorded under a custom Epi microscopy (Supplementary Note 1). The HWP was mounted on a motorized rotation stage in synchronization with camera (Fig. 1b, top). To ensure linear polarization after the dichroic filter, a Berek Polarization Compensator (BPC, New Focus^TM^) was employed (Fig. S3b). According to the truth, each rotation of the motor generates a pulse, the rising or falling edge between the two pulses could be considered as a motor period, so the modulation angle of excitation at each frame was determined by the captured frame pulse of the camera recorded synchronously. The motor rotates once and the polarization direction of excitation rotates twice, that is 4 modulation cycles, named as 4 OLID cycles (Supplementary Note 1). The lower panel in Fig. 1b shows the periodic sinusoidal response of fluorescence emission from the rotational polarized excitation (Supplementary Note 2–3). This signal can be exactly used for accurate frequency domain lock-in amplification because it carries a fixed frequency. The orientation was obtained by extracting the phase difference between the fluorescence intensity curve (Fig. 1b, bottom) and the standard modulation curve. Here the standard modulation curve is the optical response of a standard polarizer/dipole on the focal plane, and the direction of the polarizer/dipole is parallel to the *X*-axis of the imaging system (Fig. 1b, middle). Calibration of the absolute polarization angle is conducted by the standard curve (Supplementary Note 3). In the case of the significant noise or background signal in the acquisition images, as shown in Fig. 1c, we kept the zero-frequency and modulation frequency components to separate the noise and improve orientational accuracy.

### Super-resolution in OLID via polarization modulation

Fluorescent molecules in biological samples exhibit wobbling movement frequently, leading to reduced modulation contrast under rotary polarized excitation. In this case, the dipole *g*(*r*) at position *r* can be described by its polarization invariant (DC) component *g*_dc_(*r*), polarization variant (AC) component *g*_ac_(*r*), and its orientation *α*(*r*). The fluorescence anisotropy of the fluorescent dipole is defined as $p(r) = g_{\rm{ac}}(r)/(g_{\rm{ac}}(r) + g_{\rm{dc}}(r))$. We use *r*, R to describe the position of the sample plane and the camera acquisition plane, respectively. *g*(*r,θ*) and *g*(*R,θ*) are the modulation intensity of the sample intensity and the camera acquisition plane under polarization angle *θ* of excitation light respectively. *G*_dc_(*r*), *G*_ac_(*r,θ*), *G*_dc_(*R*), and *G*_ac_(*R,θ*) are the polarization invariant and polarization variant intensity of the sample plane and the camera acquisition plane by the polarization angle *θ* of excitation light respectively.

Assume the excitation intensity is *I*_0_. The emission fluorescence intensity of the excitation light with polarization direction *θ* is *g*(*r*,*θ*):

1 $$\begin{array}{{ll}} {g(r,\theta )}\, = \, {I_0 \cdot g_{\rm{dc}}(r) + I_0 \cdot g_{\rm{ac}}(r)\cos ^2(\theta - \alpha (r)),\theta \in [0^\circ ,180^\circ )} \\ \qquad\quad= \,{G_{\rm{dc}}(r) + G_{\rm{ac}}(r,\theta )} \hfill \end{array}$$

The imaging process is limited by the system’s PSF and noise, and is defined as a function *μ*(*g*(*r,θ*)). Hence, the light intensity *I*(*R,θ*) at the pixel of *R* at the camera acquisition plane can be expressed as:

2 $$\begin{array}{{ll}} {I(R,\theta )}\, = \,{\mu (g(r,\theta )) = \mathop {\sum}\limits_{r \in R} {U(r) \ast g(r,\theta )} + {\rm{noise}}} \\ \qquad\quad\,\, = \, {\mathop {\sum}\limits_{r \in R} {U(r) \ast G_{\rm{dc}}(r)} + \mathop {\sum}\limits_{r \in R} {U(r) \ast G_{\rm{ac}}(r,\theta )} + {\rm{noise}}} \\ \qquad\quad\,\, = \,{I_{\rm{dc}}(R) + I_{\rm{ac}}(R,\theta ) + {\rm{noise}}} \end{array}$$

where *U*(*r*) is the detection psf centered at *r*. *I*_dc_(*R*) and *I*_ac_(*R,θ*) are polarization invariant intensity and variant intensity at the pixel of *R* on the camera acquisition plane, respectively. In the solving process, we cannot compute the real *G*_dc_(*r*) and *G*_ac_(*r,θ*) in sample plane because of insufficient information, so we solve *G*_dc_(*R*), and *G*_ac_(*R,θ*) at pixel level and the function above can be simplified as follows:

3 $$I(R,\theta ) = \mu (g(R,\theta )) = U(R) \ast G_{\rm{dc}}(R) + U(R) \ast G_{\rm{ac}}(R,\theta ) + {\rm{noise}}$$

The solution for *G*_dc_(*R*) and *G*_ac_(*R,θ*) is represented as the following minimization problem:

4 $$\mathop {{{{{\mathrm{arg}}}}\,{{{\mathrm{min}}}}}}\limits_{G_{\rm{dc}},G_{\rm{ac}},b} {\parallel} {\mu \left( {g\left( {R,\theta } \right)} \right) - I_{\det }\left( {R,\theta } \right)} {\parallel}_{2}^{2}$$

where $I_{\det }(R,\theta )$ is the acquired modulation intensity.

We use a 3D deconvolution method in both the spatial and polarization (*x,y,θ*) domain for super-resolution reconstruction to obtain high-resolution *G*_dc_(*R*) and *G*_ac_(*R,θ*) (Supplementary Note 6). This problem is a convex and differentiable unconstrained optimization problem which can be solved by FISTA algorithm^43^ to accelerate the calculation and convergence. OLID-SDOM does not use the sparse items $(\lambda _1{{{\mathrm{|g|}}}}_1 + \lambda _2{{{\mathrm{|g|}}}}_2)$ contained in SDOM and SPoD^30^ (Eq. S2, see also Supplementary Note 7), making the reconstruction method more suitable for dense biological samples. Besides, the Poisson statistics in SDOM or SPoD are not used in OLID-SDOM because the lock-in process has the denoising effect itself (Supplementary Note 8).

### Orientation mapping with FFT phase extraction (FFTPE)

After the reconstruction mentioned above, *G*_dc_(*R*) and *G*_ac_(*R,θ*) can be obtained, which correspond to the DC signal and AC signal with modulation frequency of the dipole, respectively.

Equation 1 at the pixel level can be written as:

5 $$\begin{array}{*{20}{l}} {g(R,\theta )} \hfill & = \hfill & {\mathop {\sum}\limits_{r \in R} {\left[ {g_{\rm{dc}}(r) + \frac{{g_{\rm{ac}}(r)}}{2}} \right]} + \mathop {\sum}\limits_{r \in R} {\frac{{g_{\rm{ac}}(r)}}{2}\cos 2(\theta - \alpha (r))} } \hfill \\ {} \hfill & = \hfill & {A(R) + B(R)\cos 2(\theta - \alpha (R))} \hfill \\ {} \hfill & = \hfill & {A(R) + \frac{{B(R)}}{2}\left[ {e^{ - j2\theta }e^{j2\alpha (R)} + e^{j2\theta }e^{ - j2\alpha (R)}} \right]} \hfill \end{array}$$

Where

6 $$\left\{ {\begin{array}{*{20}{c}} {A(R) = \mathop {\sum}\limits_{r \in R} {\left[ {g_{\rm{dc}}(r) + \frac{{g_{\rm{ac}}(r)}}{2}} \right]} } \\ {B(R) = \sqrt {\left[ {\mathop {\sum}\limits_{r \in R} {\frac{{g_{\rm{ac}}(r)}}{2}\cos 2\alpha (r)} } \right]^2 + \left[ {\mathop {\sum}\limits_{r \in R} {\frac{{g_{\rm{ac}}(r)}}{2}\sin 2\alpha (r)} } \right]^2} } \end{array}} \right.$$

According to the cosine squared form $2\pi f_{\bmod }\theta = 2\theta$ in Eq. 5, the modulation frequency *f*_mod_ is 1/π. We define the number of OLID periods participating in the calculation as *m*, the sampling point during each OLID period as *M*, the total number of sampling points as *N* = mM, and the variable of modulated angle as Δ*θ*. The direction of polarization of the excitation light changes *π*. during each OLID period, so *M* = *π/Δθ* and the modulation angle is *θ* = *nΔθ,n* = 0,…,*N*-1. Therefore, Eq. 5 can be written as:

7 $$g(R,\theta ) = A(R) + \frac{{B(R)}}{2}\left[ {e^{ - j2n\Delta \theta }e^{j2\alpha (R)} + e^{j2n\Delta \theta }e^{ - j2\alpha (R)}} \right]$$

Take the *N* point Fourier transform of *g*(*R*,*θ*),

8 $$\begin{array}{lll} {G(R,k)} &=& {\mathop {\sum}\limits_n^{N - 1} {g(R,\theta )e^{ - j\frac{{2\pi kn}}{{mM}}}} } \\ &=& {\mathop {\sum}\limits_n^{N - 1} {g(R,\theta )e^{ - j\frac{{2kn\Delta \theta }}{m}}} } \\ &=& {\mathop {\sum}\limits_n^{N - 1} {\left\{ {A(R) + \frac{{B(R)}}{2}\left[ {e^{ - j2n\Delta \theta }e^{j2\alpha (R)} + e^{j2n\Delta \theta }e^{ - j2\alpha (R)}} \right]} \right\}}}\\& &e^{ - j\frac{{2kn\Delta \theta }}{m}} ,k = 0,1,...,N - 1 \end{array}$$

The zero-frequency component is $G(R,0) = \mathop {\sum}\nolimits_n^{N - 1} {A(R)}$, and the inverse Fourier transform of *G*(*R*,0) gives *G*_dc_ (*R*).

The *G*_ac_ (*R,θ*) solved is the component of modulation frequency 1/π, where *k* = *m*,

9 $$\begin{array}{l}G(R,m) = \mathop {\sum}\limits_n^{N - 1} {A(R)e^{ - j2n\Delta \theta }} + \mathop {\sum}\limits_n^{N - 1} {\frac{{B(R)}}{2}e^{ - j2n\Delta \theta }e^{j2\alpha (R)}e^{ - j2n\Delta \theta }}\\ \qquad \quad \qquad\ + \mathop {\sum}\limits_n^{N - 1} {\frac{{B(R)}}{2}e^{j2n\Delta \theta }e^{ - j2\alpha (R)}e^{ - j2n\Delta \theta }} = \frac{N}{2}B(R)e^{ - j2\alpha (R)}\end{array}$$

The phase of the (*m* + 1)_th_ point of the Fourier transform can be extracted to obtain the orientation information and to conduct orientation mapping. The inverse Fourier transform of *G*(*R,m*) gives $G_{ac}(R,\theta ) = N \cdot B(R)e^{j2(\theta - \alpha (R))}/2$. *A*(*R*) and B(*R*) can be calculated thereafter. Orientation uniform factor (OUF)^32^ is defined as $OUF = 2B(R)/(A(R) + B(R))$, representing the anisotropy of each pixel. The closer to 1 the OUF is, the more uniform the orientation of dipoles keeps, while the closer to 0, the more random the molecules are.

The single-point simulations were used in Supplementary Note 4 to verify that the effect of photobleaching on the measurement accuracy and error is negligible. In Fig. S4, the orientation accuracy extracted from 4 OLID periods would be two times higher than that extracted from 1 OLID period, and the OUF accuracy could be increased by 1.6 times (OUF = 0.1), with reduced OUF error. The simulations in Supplementary Note 5 demonstrated the performance of the OLID-SDOM. As shown in Fig. S5a–b, a worse signal-to-noise ratio (SNR) in raw data resulted in artifacts on the reconstructed image with weaker OUF and orientation measurement precision, but the resolution would not deteriorate. In addition, the precision of orientation of OLID-SDOM could reach 4.7° (Fig. S5a). In Fig. S5c, the lowest resolution was 200 nm (OUF = 1.0, Δ*α* = 0°), and two points with a spatial distance of 80 nm could be separated (OUF = 1.0, Δ*α* = 90°). This is understandable because the dipole angular difference provides further “distance” at the spatial-angular dimension (Supplementary Note 6). Referred to the orientation mapping (Fig. S5d), the distance of 140 nm could be distinguished when Δ*α* = 10° and the distance of 50 nm could be distinguished when Δ*α* = 90°.

The flowchart of OLID-SDOM is shown in Fig. 1d. The discrete Fourier transform in time (or *θ*) axis was carried out in the multi-period data. The modulation frequency component and background component were retained to generate single-period data. We called this process OLID, which could enhance the image SNR by 10 dB (Supplementary Note 5). After OLID, the 3D deconvolution was implemented to reconstruct *G*_dc_(*R*) and *G*_ac_(*R*,*θ*). Then, after the phase information was extracted, the orientation mapping was obtained. Compared with SDOM (Fig. 1e), OLID-SDOM has the following advantages: (1) the preprocessing of OLID reduces the influence of noise, and speed up the convergence; (2) the sparsity constraint is avoided in OLID-SDOM, comparing with that in the SDOM process; (3) 3D deconvolution of OLID-SDOM has a higher resolution than the 2D deconvolution of SDOM (Supplementary Note 6); (4) the orientation mapping method by FFTPE is faster than the least-squared fitting of SDOM with similar or higher accuracy (Supplementary Note 4).

**Fig. 2:**
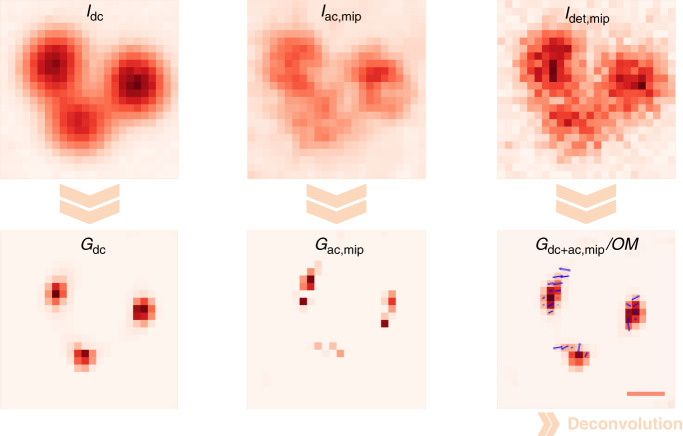
Super-resolution imaging of 120-nm DNA origami with OLID-SDOM. The reconstruction process of OLID-SDOM is illustrated by the experimental results of DNA origami, which contains two neighboring fluorophores with a distance of 120 nm at both ends. Multi-frame images are displayed by maximum intensity projection with subscript *mip* in the label. *I*_det_ is decomposed into *I*_dc_ and *I*_ac_, and *G*_dc_ and *G*_ac_ can be obtained by deconvolution of the *I*_dc_ and *I*_ac_, respectively. By combining the *G*_dc_ and *G*_ac_ and calculating the orientation mapping (labeled with OM) according to FFTPE, $G_{{{{\mathrm{dc}}}} + {{{\mathrm{ac}}}}}/{{{\mathrm{OM}}}}$ is obtained. The fluorophores can be resolved from the intensity image of super-resolution *G*_ac,mip_ image or from the super-resolution orientation mapping image $G_{{{{\mathrm{dc}}}} + {{{\mathrm{ac,mip}}}}}/{{{\mathrm{OM}}}}$. The dipole orientations are indicated by blue arrows imposed on the image. Scale bar: 200 nm

In order to illustrate further how OLID-SDOM works, DNA origami with two ATTO647N fluorophores with a distance of 120 nm at both ends was imaged by OLID-SDOM (Fig. 2). The two emitters could be only distinguished by the *G*_ac,mip_ image, but not be revealed by wide-field or reconstructed super-resolution *G*_dc_ image, or by the orientation-mapping image $G_{{{{\mathrm{dc}}}} + {{{\mathrm{ac,mip}}}}}{{{\mathrm{/OM}}}}$.

### OLID-SDOM imaging of subcellular organelles

Subcellular organelles, such as the plasma membrane, endosomes, lysosomes, mitochondria, the Golgi, and the endoplasmic reticulum (ER), perform a variety of functions in eukaryotic cells. Their membranes are folded into various shapes to accommodate different functions, often with highly curved morphologies and nanometer-scale dimensions^44^. In addition, the dipole orientation of fluorophores attached to the organelle provides insights into the molecular formation, transformation, and dynamics of the organelles. However, the applications of FPM are limited in the research of subcellular structures due to various reasons: (1) The sizes of most subcellular organelles are below the diffraction-limited resolution (200 nm); (2) There is low polarization modulation of the subcellular organelles due to the averaging of polarization signals within the neighboring area; (3) Single molecular imaging FPM techniques such as Polar-dSTORM^29^ could not be applied to live cell imaging due to slow imaging speed. By contrast, OLID-SDOM is able to provide highly sensitive measurement of the anisotropy of subcellular organelles with high imaging speed, thus bringing potential solution to these problems.

**Fig. 3:**
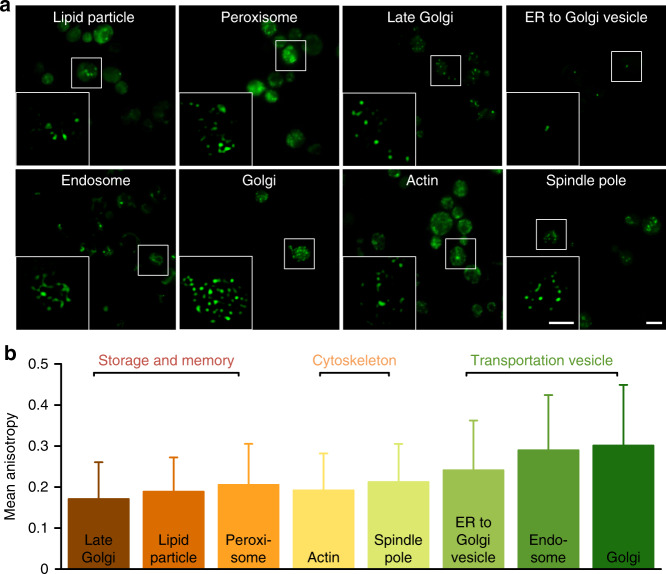
OLID-SDOM imaging of subcellular organelles in live budding yeast cells. **a** Wide-field epifluorescence microscopy images of GFP labeled subcellular organelles, such as lipid particles, late Golgi, actin, spindle pole, peroxisome, ER to Golgi vesicle, Golgi complex, and endosome. Scale bar: 5 μm. The diagrams inset is the *G*_ac_ of reconstructed OLID-SDOM images of the white boxed area. Scale bar: 2 μm. **b** Anisotropy distribution of different subcellular organelles indicates that subcellular organelles, which have a different function in cells, have specific OUF.

To demonstrate the widespread application of OLID-SDOM, we imaged eight different subcellular organelles of live budding yeast cells - a model system in cell biology. Each subcellular organelle in yeast was encoded with specific GFP-tagged proteins, such as GFP-ERG6 in lipid particles, GFP-CHC1 in late Golgi, GFP-SAC6 in actin, GFP-SPC42 in spindle pole, GFP-PEX3 in peroxisome, GFP-SEC13 in ER to Golgi vesicle, GFP-ANP1 in Golgi complex, and GFP-SNF7 in endosome^45^. The orientation of GFP fused with proteins can represent the actual orientation of tagged proteins^13^. Compared to the conventional wide-field image, OLID-SDOM allowed better visualization of mitotic spindles and microtubules with higher resolution (Fig. 3a). The spindle pole body protein Spc42, which localizes as one diffraction-limited spot in wide-field, could be resolved to an elongated body.

Next we suppose that different subcellular organelle might have specific OUF values and standard deviation (Fig. 3b). Surprisingly, we found that transportation vesicles have the largest OUF, while the standard deviation of the OUF value in the spindle is accordingly larger than that of other organelles. The transportation vesicles are known to frequently undergo fusion and fission^46^, indicating that the binding force between the bio-macromolecule and the dye molecule is highly anisotropic. Furthermore, the OUF of cytoskeletons, such as actin and spindle pole, is smaller. Not only that, in response to the storage and memory, the vesicles, such as lipid and late Golgi, have the smallest OUF, though we also noticed that the peroxisome has a stronger modulation. Therefore, these results indicate that OUF can indeed be an indicator of whether subcellular organelles can function normally. Abnormal OUF can be a sign for abnormal cells (for example, cancer cell). This interesting observation may add another dimension for understanding the organelle dynamics and expanding our toolset for cell biology research and even for clinical diagnosis and early cancer screening.

### Dual-color OLID-SDOM imaging of biological filaments

Essential cellular functions as diverse as genome maintenance and tissue morphogenesis rely on the dynamic organization of filamentous assemblies. The precise spatial arrangement of cytoskeletal protein filaments is the key to mechanical force generation driving animal tissue morphogenesis. Cellular cytoskeleton, such as actin^18,19,29^, microtubule^20^, or septin^21^, is another type of structure displaying intense fluorescence polarization. FPM could extract the structural organization of biological filaments by determining the orientation of fluorescent labels. However, as the measuring accuracy is also limited by optical diffraction, SDOM is unable to discriminate between the intrinsic orientational mobility of the fluorophore labels and the real structural disorder of the labeled biomolecules, whereas OLID-SDOM can tanks to its amplified sensitivity.

**Fig. 4:**
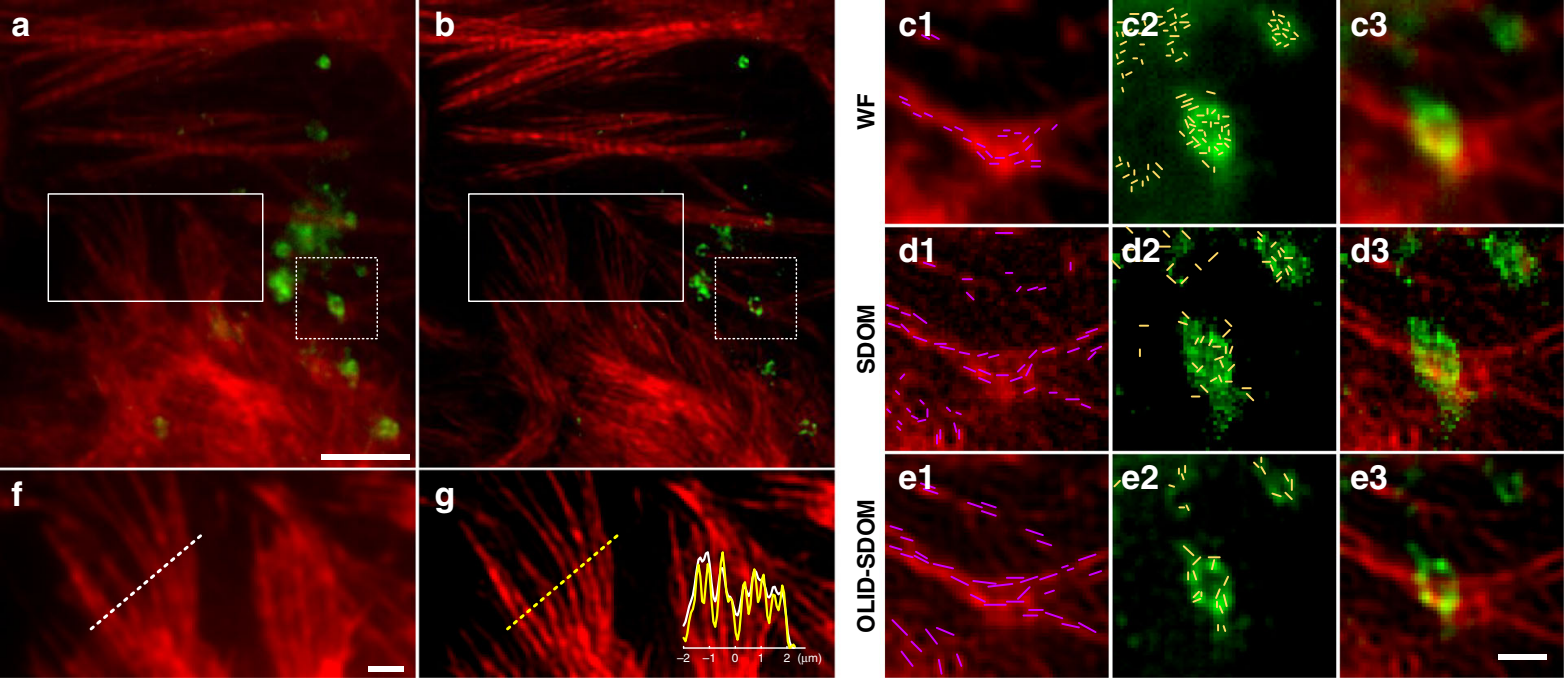
Dual-color OLID-SDOM imaging of lysosomes and actin in U2OS cells. **a**, **b** Conventional wide-field and OLID-SDOM image of Alexa Fluor 568 phalloidin labeled F-actin (red), and GFP-lamp1 labeled lysosome (green). Scale bar: 5 μm. **c**–**e** Expanded view of the dual-color wide-field image, SDOM image, and OLID-SDOM image of F-actin (red) and lysosome (green) of the white dotted box in (**a**–**b**) Scale bar: 1 μm. The direction of the carmine and yellow lines denotes the dipole orientation of F-actin and lysosome, respectively. **f** and **g** show the expanded view of wide-field and super-resolution images of transfected actin filaments of the white box in (**a**–**b**), respectively. The inset shows the normalized intensity profiles taken from the regions indicated in (**f**) and (**g**). Scale bar: 1 μm

Here, we labeled F-actin and lysosomes for dual-color super-resolution imaging. Actin filaments play an important role in cellular pathways^47^ and display high-density and strong fluorescence polarization. Lysosomes participate extensively in many critical cellular processes such as apoptosis, energy production, and cellular metabolism, presenting with weak intensity and fluorescence polarization. Studies showed that actin filaments were associated with the lysosome movement and docking, i.e., the delivery of internalized molecules to lysosomes^48^, which could be seen from the position relationship of the two organelles in Fig. 4c–e. Individual actin filaments and their organization could not be resolved in the conventional wide-field image due to diffraction limit (Fig. 4a), but could be clearly resolved in OLID-SDOM image (Fig. 4b). In the magnification region, much denser structures were resolved by SDOM and OLID-SDOM than by wide-field (Fig. 4c1, d1, e1), while more artifacts were generated by SDOM algorithm. Although in some regions (Fig. 4d1, e1), the actin filaments were too dense to be resolved clearly in super-resolution images, the orientation information along actin direction could help resolve those details. However, for low SNR and weakly polarized organelles like lysosome, the artifacts from SDOM has drowned out the sample signal, make it impossible to distinguish different objects. Furthermore, orientation mapping was disordered (Fig. 4d2, d3). In the meantime, OLID-SDOM showed the reconstruction ability of samples with low SNR and low anisotropy, and the structure and the orientation information could be obtained (Fig. 4e2, e3).

### The dynamic behavior of the mitochondria can be visualized by live-cell OLID-SDOM

**Fig. 5:**
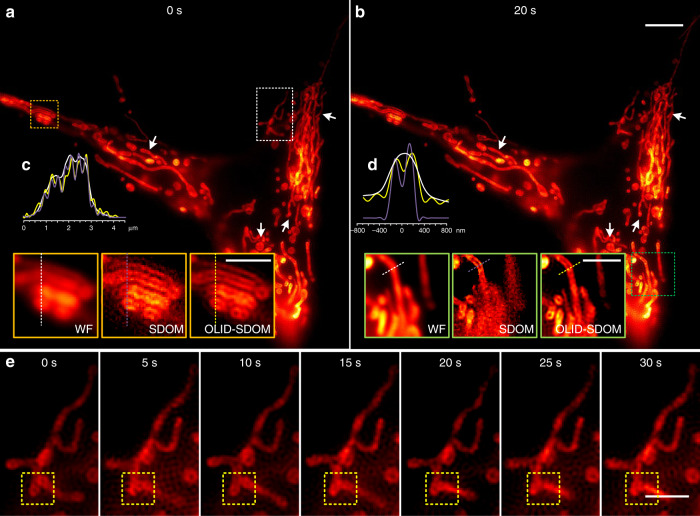
Dynamic super-resolution live-imaging of TOM20-GFP labeled mitochondria in U2OS cells. **a**, **b** Individual frames showed the dynamic behavior of the mitochondria. The corresponding white arrow in both (**a**) and (**b**) figured out subtle morphologic changes over 20 s. Scale bar: 5 μm. **c** Enlarged wide-field image, SDOM image and OLID-SDOM image of yellow dotted box in (**a**), and the inserted profiles showed the normalized intensity profiles taken from the dashed lines indicated in images with the yellow box. Scale bar:2 μm. **d** Enlarged wide-field image, SDOM image and OLID-SDOM image of green dotted box in (**b**), and the inserted profiles showed the normalized intensity profiles taken from the dashed lines indicated in images with the green box. Scale bar: 2 μm. **e** Representative time-lapse images of sub-region of white dotted box in a illustrated the remodeling and growth of sub-mitochondria at indicated time points with higher magnification. Scale bar: 2 μm

The ability of OLID-SDOM to produce super-resolution images with a temporal resolution of 100 f/s (Supplementary Note 2) allows the detailed imaging of highly dynamic cellular processes. We demonstrated this versatility by super-resolving the dynamics of mitochondria in U2OS cells transfected with TOM20 fused to GFP. Mitochondria are pleomorphic organelles with structural variations depending on cell type, cell-cycle stage, and intracellular metabolic states in eukaryotic cells. Mitochondria play a pivotal role in energy production, apoptosis, and cell metabolism. The morphology of mitochondria is highly variable depending on the organelles’ functioning. However, few studies of the rapid dynamics of mitochondria involving their super-resolution morphology have been reported. The speed of OLID-SDOM enables capturing the millisecond-scale remodeling and growth of the sub-mitochondrial. As shown in Fig. 5, numerous rod-like, reticulum, and granular mitochondria were observed (Fig. 5). In higher magnification sub-regions, OLID-SDOM can distinguish more structural details than wide-field images, and has similar performance to SDOM at the sparse position but fewer artifacts than SDOM at dense position (Fig. 5c, d). Figure 5e showed typical super-resolved time-lapse image of mitochondria over 20 s. The process of mitochondrial fusion and fission could be visualized directly in yellow dashed boxes. Studies showed that changes in mitochondrial morphology are the main cause of a variety of viral human neurodegenerative diseases^49,50^. Therefore, OLID-SDOM provides a new tool to detect diseases, and offers new insight into the dynamic behavior of mitochondria in vivo, signifying further molecular mechanisms that morphology can function as an indicator of cell states.

### Time-lapse OLID-SDOM microscopy reveals the morphology and orientation dynamics of dendritic spines in live neurons

**Fig. 6:**
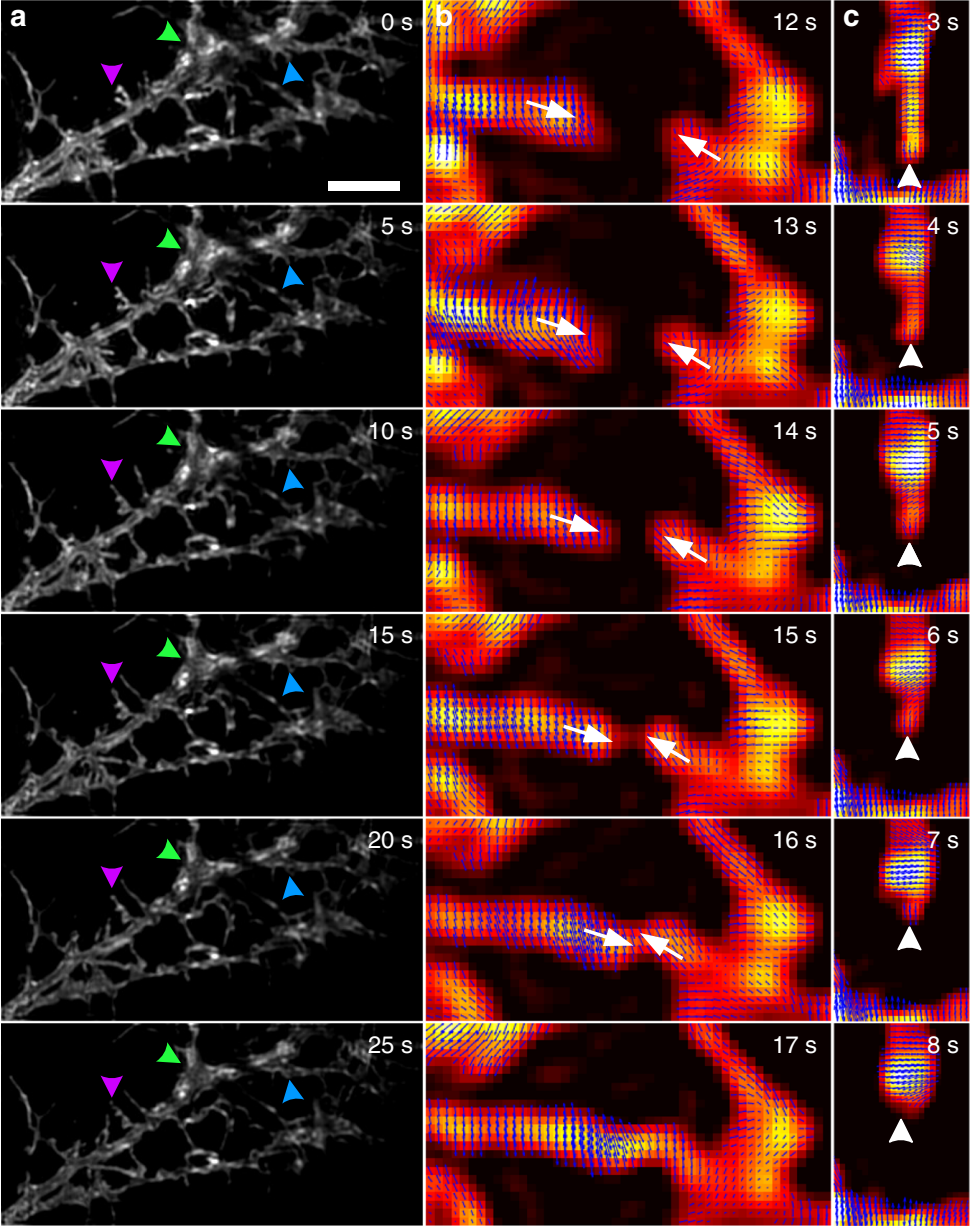
In vivo OLID-SDOM imaging of dendritic spines stained with lipophilic tracer DiI in live hippocampal neurons. **a** Individual OLID-SDOM frames of dendritic spines from super-resolution time-lapse imaging. The small triangles denote subtle morphologic changes. Scale bar: 5 μm. The triangles of each color indicate the change of the corresponding position structure within 25 s. **b** Orientation mapping of two spines in extending and joining process. The white arrows display the direction of extension of the structure at the corresponding position. **c** Morphology and orientation dynamics of a shrinking spine. The white triangles display the contraction direction of the corresponding position. The direction and length of blue arrow in each pixel indicate the orientation and OUF of the corresponding pixel

Dendritic spines are the active building blocks of the neuron network in the brain. The spines are responsible for memory storage, synaptic transmission, and establishing connections between neurons^51,52^. The study of the highly dynamic formation and dissociation of dendritic spines can help us to understand the synaptic functions. Previously, STED super-resolution microscopy has been employed to study the formation of dendritic spine^53^. To observe both the morphological plasticity and polarity of dendritic spines, here we reported the dynamic imaging of dendritic spines with a sub-diffraction resolution of 140 nm at 100 f/s frame rate using the frame-rolling method (Supplementary Notes 2, 5) in living neurons, using OLID-SDOM techniques (Fig. 6a). Morphological changes in shape and size of dendritic spines were clearly visualized in the six successive reconstructed super-resolution images (Fig. 6) from time-lapse images which last 30 s long (see Supplementary Movie S1–S2). Features <100 nm such as the thickness of the spine neck or subtle changes in the shape of the spine head, for example, from thin strip shape to mushroom shape-could be measured well by OLID-SDOM. Moreover, we found that the polarization of spines provides additional information in understanding the behaviors of their spines. For example, Fig. 6b documented the forming of the connection between two spines growing toward each other, in which the polarization angles of both leading edges were toward each other. In Fig. 6c, during a “kiss-and-run” touch to the nearby dendritic, the growth of the spine head even made the polarization direction perpendicular to that of the body. Interestingly, the first bud didn’t connect to the dendritic; instead, its neighboring bud extended and reached the destination (Supplementary Movie S3). Because the polarization reflects the force that applies to the membrane^18^, OLID-SDOM may be applied extensively to study synaptic activities.

Figure 6b illustrated the subtle anisotropy changes of an extended spine upon the reconstructed super-resolution image. In general, structural morphology dynamics were impossible to be visualized below the resolution of the optical system. OLID-SDOM extracted the orientation information faithfully, offering finer details of super-resolved dendritic structure. By precisely mapping the orientation dynamics along each imaged dendrite, we found that higher orientation dynamics are in agreement with the higher frequency of local morphology changes. Neuron dendrite morphogenesis is critical to structural plasticity and memory mechanisms. Remarkably, the orientation was almost invariable in the morphology-still region. However, in spine-extending regions, the orientation changed rapidly along the extending direction. Studies showed that the actin cytoskeleton interacted with plasma membrane changes the morphology^54^. OLID-SDOM techniques may even drive forward the studies of synaptic activity regarding to the dynamics of dendritic spine morphology plasticity.

The principle of OLID-SDOM super-resolution via polarization demodulation is also suitable for three-dimensional imaging. With the z-scanning and polarization modulation applied during each frame acquirement, the 3D super-resolution intensity imaging could be achieved with the in-plane fluorescent dipole orientation measurement. Benefitted from the resolution enhancement of OLID-SDOM, the shell structure of a 1 µm diameter fluorescent bead could be clearly resolved (Fig. S10a). Fig. S10c showed the super-resolution 3D spatial distributions of the actin bundles of HeLa cell with the depth of actin color-coded, and Fig. S10b showed three crossing actin fiber bundles, with the upper two perpendiculars to the plane and the lower one remaining in the plane (see also Supplementary Note 9).

## Discussion

In this work, we report OLID-SDOM, which can attain super-resolution of low SNR samples through extracting the ensemble dipole orientation. OLID has been employed to significantly enhance the signal from the noise (Supplementary Notes 4, 8), as the dipole orientation is relatively weak in live cells. Taking advantage of the enhanced dipole orientation, DNA origami with a distance of 120 nm can be clearly resolved. Combined with orientation mapping, the OLID-SDOM in simulation can distinguish the minimum lateral spatial distance of 50 nm. Furthermore, OLID-SDOM provides more additional insight to recognize precisely the orientation information of the super-resolved subcellular structures. We have examined the modulation factors of various subcellular organelles, such as lipid particles, late Golgi, actin, spindle pole, peroxisome, ER to Golgi vesicle, Golgi, and endosome in budding yeast. The strong modulation indicates their binding affinity to the dye, which can also be reflected from their cellular functionality: the OUF of transportation vesicles is higher than that of the actin filaments whose visualization using fluorescent markers is an essential tool for getting a deeper understanding of the structural cytoskeletal dynamics, and the storage and memory organelles are generally with weaker modulation than transportation vesicles. Dual-color OLID-SDOM image of lysosomes and actin demonstrates their both super-resolved structures and orientations of these two complexes. Finally, this system was employed to study the dynamic plasticity of the dendritic spines, indicating the orthogonal orientation of the lipid molecules alongside the direction of dendritic spine formations, in accordance with the published observation^54^.

Moreover, the OLID can be applied in other imaging techniques, such as SIM and SMLM (Single-Molecule Localization super-resolution Microscopy), to preprocess the original images, thus improving the reconstruction accuracy and quality. In contrast to SDOM, the OLID-SDOM has fewer artifacts in the condition of low SNR and low anisotropy, and higher calculation speed for orientation mapping and image reconstruction.

OLID-SDOM has revealed the orientation of subcellular organelles and spines with sub-diffraction-limited spatial resolution, and with a temporal resolution of 100 f/s and orientation precision of 4.7°(Fig. S5a) and the ability in 3D super-resolution imaging. OLID-SDOM can be employed to image mitochondrial morphology in human neurodegenerative diseases, thus has the potential to be applied in clinics and hospitals. Together with OUF, OLID-SDOM could become new tools for the detection and early screening of abnormal or even cancer cells for the early diagnosis of diseases and cancer. Benefitted from its simple hardware setup, low illumination power (~200 W/cm^2^), high-speed imaging capability, high SNR, and compatibility to various dyes and fluorescent proteins, we anticipate that OLID-SDOM will hold great potential in the next generation super-resolution live-cell microscopy.

## Materials and methods

### Sample preparation

The specimen slide of Alexa Fluor 561 labeled F-actin in the mouse kidney section was purchased from Invitrogen (Cat. No. F24630). DNA origami sample was purchased from GATTAquant (GATTA-STED NANORULER 120 nm, http://www.gattaquant.com/). The two ends of the NANORULER were marked by the dense arrangements of ATTO647N.

Organelle imaging in live budding yeast cells: All strains of subcellular organelles were bought from the genome-wide GFP-tagged yeast protein localization database^45^ (Life Technology, Cat. No. 95702). Appropriate budding yeast strains were selected from the commercial Yeast GFP Clone Collection (Thermo Fisher Scientific, https://clones.thermofisher.com/cloneinfo.php?clone=yeastgfp) in −80 °C, placed to the YPD agar (1% yeast extract, 2% peptone, 2% glucose, and 2% agar), and cultured in an incubator at 30 °C about 2–3 days. 5 mL SD-His medium (0.17% Yeast Nitrogen Base, 0.5% (NH_4_)_2_SO_4_, 2% glucose, DO Supplement –His) and a small amount of yeast clones from the YPD agar were added into the centrifuge tube, and sealed. Clones was incubated in a shaker at 30 °C, 180 rmp for 16 h until the exponential period. 170 μL SD-His agar was dropped onto one glass slide, and pressed to solidification with another glass slide. Then two glass slides were separated and a block on one slide was left. The cell suspensions (10 μL) were poured over the YPD block, then the coverglass was covered and the plate was sealed.

#### Dual-color

U2OS cells were seeded on coverglass in DMEM phenol red-free media (GIBCO BRL, Grand Island, NY) supplemented with 10% fetal bovine serum (FBS) at 37 °C in a 5% CO_2_ incubator. Cell Light reagent (Invitrogen^TM^, Cat. No. C10507) was added and cells were cultured for 24 h. Then the cells were moved to microscopy, to imaged the transfected cells expressing the Lamp1-GFP-tagged lysosome. Next, the cells were washed twice with pre-warmed phosphate-buffered saline (PBS, pH 7.4) and fixed with 3.7% paraformaldehyde (PFA) in PBS for 10 min at room temperature. The fixed samples were washed twice with PBS. The supernatant was suctioned, the prepared dye (1 mL PBS: 25 μL Alexa Fluor 568 phalloidin of the 40× methanol stock solution, Cat. No. A12380) was added, and the cells were incubated for 1–2 h away from light. Finally, the samples were washed three times with PBS, and the plate was sealed.

#### Live cell mitochondria imaging

U2OS cells were plated on eight-well chambered coverglass (LabTek II, Cat. No. 155409) in DMEM supplemented with 10% FBS at 37 °C in a 5% CO_2_ incubator. CellLight™ Mitochondria-GFP reagent was added and cells were cultured for 24 h. Thereafter the clels were moved to microscopy for dynamic super-resolution imaging.

#### Live hippocampal neurons imaging

Hippocampal neuron cells were plated on four-well chambered coverglass (LabTek^TM^ II, Cat. No. 155382) bottoms in a culture medium at 37 °C in a 5% CO_2_ incubator. The cell culture medium was Neurobasal-A medium (Gibco, Cat. No. 10888022) containing 10 mM d-glucose (Gibco, Cat. No. A2494001), penicillin/streptomycin (Invitrogen, Cat. No. 15140163), B27 supplement (Gibco, Cat. No. 17504044) and GlutaMAX (Gibco, Cat. No. 35050061). The DiI stain (Invitrogen™, Cat. No. D282) was diluted to 1 μM in the culture medium. The liquid was removed in the chamber, the prepared DiI staining medium was added, and the cells were cultured in an incubator at 37 °C for 20 min. The cells were washed by adding a fresh warm growth medium and incubated at 37 °C for 5 min. This wash step was repeated two more times, and then the cells can be imaged in the culture medium.

## Supplementary information

Supplementary Information for “Polarization Modulation with Optical Lock-in Detection Reveals Universal Fluorescence Anisotropy of Subcellular Structures in Live Cells”

Widefield video of dendritic spines in live hippocampal neurons

OLID-SDOM video of dendritic spines

Morphology and orientation dynamics of a shrinking spine

## Data Availability

The OLID-SDOM software and datasets are openly available from Github (https://github.com/merlinguan/OLID-SDOM).
