# Supplementary material for: Polarization modulation with optical lock-in detection reveals universal fluorescence anisotropy of subcellular structures in live cells
Source: Light Sci Appl. 2022 Jan 1;11:4. doi: 10.1038/s41377-021-00689-1 (PMC8720311; doi:10.1038/s41377-021-00689-1)
Supplement: Supplementary file 1 — Supplementary Information for “Polarization Modulation with Optical Lock-in Detection Reveals Universal Fluorescence Anisotropy of Subcellular Structures in Live Cells” [file 41377_2021_689_MOESM1_ESM.docx]

**Supplementary Information for “Polarization Modulation with Optical Lock-in Detection Reveals Universal Fluorescence Anisotropy of Subcellular Structures in Live Cells”**

Meiling Guan^1,*^, Miaoyan Wang^1^,*, Karl Zhanghao^1,2^,*, Xu Zhang^3,4,8^, Meiqi Li^1^, Wenhui Liu^4^, Jing Niu^3^, Xusan Yang^1^, Long Chen^3,4^, Zhenli Jing^3^, Micheal Q Zhang^5,3,7^, Dayong Jin^2,6^, Peng Xi^1,2,9^ , Juntao Gao^3,4,†^

^1^ Department of Biomedical Engineering, College of Future Technology, Peking University, Beijing 100871, China.

^2^ UTS-SUStech Joint Research Centre for Biomedical Materials & Devices, Department of Biomedical Engineering, College of Engineering, Southern University of Science and Technology, Shenzhen, Guangdong, China

^3^ MOE Key Laboratory of Bioinformatics; Bioinformatics Division, Center for Synthetic & Systems Biology, BNRist; ^4^ Center for Synthetic & Systems Biology; Department of Automation, Tsinghua University, Beijing 100084, China

^5^ Department of Biological Sciences and Center for System Biology, The University of Texas at Dallas, Richardson, 75080, USA

^6^ Institute for Biomedical Materials and Devices (IBMD), Faculty of Science, University of Technology Sydney, NSW, 2007, Australia.

^7^ School of Medical Sciences, Tsinghua University, Beijing 100084, China

^8^ Beijing Institute of Collaborative Innovation, Beijing 100094, China.

^9^ National Biomedical Imaging Center, Peking University, Beijing 100871, China

*These authors contributed equally to this work

†Email: [jtgao@tsinghua.edu.cn](mailto:jtgao@tsinghua.edu.cn)

**Outline**

Supplementary Note 1: System setup

Supplementary Note 2: Synchronization

Supplementary Note 3: Polarization angle calibration

Supplementary Note 4: Performance of FFT phase extraction in simulation

Supplementary Note 5: Performance of OLID-SDOM in simulation

Supplementary Note 6: Comparison between 2D deconvolution and 3D deconvolution

Supplementary Note 7: Comparison between SDOM, SPoD and OLID-SDOM

Supplementary Note 8: Denoising of polarization modulation data

Supplementary Note 9: 3D Imaging with OLID-SDOM

References

# Supplementary Note 1: System setup

The experimental setup of OLID-SDOM was based on a home-built epi-illumination microscope. As shown in **Fig. S1**, the excitation light sources were polarized continuous wave lasers emitting at 488, 561, and 647 nm (Coherent QIBS), with incident power of ~20 mW before the objective. The lasers were reflected by the dichroic mirrors (DM1 and DM2), and then passed through a half-wave plate (HWP) mounting on a motorized rotation stage, rotating at a given frequency. The incident polarization angle rotated at twice the rate of HWP. A polarization compensator was placed on the optical path to keep the light linearly polarized. After beam expanding, the excitation light was reflected by a dichroic mirror (DM3) and focused on the objective back focal plane by a long focal distance lens (L3, f = 400 mm). Epi-illumination was demonstrated by an oil-immersion objective lens, focusing light on the sample plane. The same objective collected the emitted fluorescence and passed through the DM3 and tube lens, before reaching the charge coupled device (CCD) camera.

**Fig. S1. Experimental Setup**. M: mirror. DM: dichroic mirror. HWP: Half wave plate mounted on a rotation motor. BPC: Berks polarization compensator. L: lens. OBJ: objective. F: filter.

# Supplementary Note 2: Synchronization

The camera was controlled by MicroManager software^1^. LabVIEW^2^ software and myDAQ (National Instruments) were used to acquire the motor rotation pulse, synchronizing with the camera acquisition pulse. The motor rotated 360$\text{°}$ for one cycle, and the polarization angle rotated 720$\text{°}$. Therefore, there were four modulation periods (180$\text{°}$ per period) in one rotation circle.

Given a fixed signal-to-noise ratio (SNR) and random orientation angles, modulated signals with an OUF () interval of 0.1 were generated by adding Gaussian white noise and Poisson noise. Under each condition, 1000 tests were repeated. The measurement error of OUF = (calculation OUF – preset OUF)/ preset OUF, and the measurement precision of orientation was the standard deviation of the measurement error of orientation. As shown in **Fig. S2**, with the increase of the number of sampling points per OLID period, the calculation error of OUF decreased, and the accuracy remained unchanged, while the calculation accuracy of orientation improved. Regardless of fluorescence intensity, the following was the analysis of the temporal resolution.

The highest acquisition frame was 100 fps for sCMOS, and the shortest exposure time was 1 s/100=10 ms. The maximum motor speed was set to 200 rpm (Revolutions Per Minute), so the minimum cycle of motor rotation was 0.3 s (60 s/200 rpm).

Referring to the SNR of the acquired images in our experiment, we set the SNR to 20 dB and 30 dB, and the calculation error of OUF and orientation accuracy were acceptable in any case of the number of sampling points. At this time, the time resolution was mainly limited by the motor speed, which means that one OLID period is 0.3 s/4 = 75 ms, and we used 4 OLID periods for reconstruction. Therefore, if we used period-rolling (period: 1-4, 2-5, 3-6,…) method to reconstruct, the highest frame could reach 13.3 fps (1 s/75 ms = 13.3 fps, less than 7.5 sample points per OLID period). If we used frame-rolling (frame: 1-16, 2-17, 3-18, 4-19,…) method to reconstruct, the highest frame could reach 100 fps (1 s/10 ms = 100 fps, more than 7.5 sample points per OLID period).

**Fig. S2. The calculation error and accuracy varied with sampling points within each OLID period**. The left column is the relationship between the calculation error of OUF and the number of sampling points per OLID period in the case of different OUF. The right column is the relationship between the calculation accuracy of orientation and the number of sampling points per OLID period in the case of different OUF. From the first row to the second row, every row is the simulation result in the condition of the SNR of 20 dB and 30 dB, respectively.

# Supplementary Note 3: Polarization angle calibration

We usually set a time period to collect 10 pictures. ~40 pictures were collected by our CCD with one revolution of the motor. The imaging speed is mainly determined by the EMCCD exposure time, and the exposure time is mainly set according to the property of the fluorophore, so as to ensure the acquired images have a relatively high SNR. The rotation speed was adjusted according to the camera acquisition speed. For reconstruction, the DNA origami was imaged with 10 lock-in periods and 100 ms exposure time, by Evolve 512 Delta EMCCD (Photometrics). Other samples were imaged with 2 ~ 4 lock-in periods and 25 ms exposure time, by Zyla 4.2 sCMOS camera (Andor, Oxford Instruments).

**Fig. S3.** (a) The sync signals of camera readout and motor rotation. (b) The polarization state of the modulated light behind the objective in different directions before (upper) and after (lower) polarization compensation.

A standard polarizer/dipole was put on the focal plane, of which the direction was parallel to the X-axis of the imaging system. An optical dynamometer was used to detect the laser intensity through the polarizer. The laser was modulated to a cosine squared curve. As shown in **Fig. 3a**, the motor rotated 360$\text{°}\text{ }$ for one cycle, with the polarization angle rotating 720$\text{°}$. Therefore, there were four modulation periods in one rotation circle. Thereafter, we set the rising edge of the rotating pulse to ‘0’ degree and set the corresponding angle in cosine squared curve as the calibration angle. The calculated polarization angle was the sum of the calibration angle and the rotation angle.

# Supplementary Note 4: Performance of FFT phase extraction in simulation

The performance of FFT phase extraction (FFTPE) in OLID-SDOM, containing the angular measurement accuracy and the OUF calculation error, was simulated here. We implemented the single-point simulation to demonstrate the error of OUF and the accuracy of orientation in different period number and OUF.

Given a fixed OUF and random orientation angles, we generated the modulated signals, added white noise with SNR=20 dB and Poisson noise, and repeated this experiment 1000 times. The normalized error of OUF and measurement error of orientation by different OLID cycles were counted thereafter. The normalized error of OUF was defined as: (calculation OUF – preset OUF)/ preset OUF, and the measurement error of orientation was: calculation (orientation – preset orientation). According to **Fig. S4**, with the condition of low OUF, the more OLID period was, the higher the measurement accuracy of orientation would be. Among them, the orientation accuracy extracted from 4 OLID periods would be about 2 times higher than that extracted from 1 OLID period, and the OUF accuracy could be increased by 1.6 times (OUF = 0.1), with reduced OUF error. For the sake of photobleaching, we simulated the photobleaching process using an exponential decay function, and other steps were the same as before. **Fig. S4** shows that the effect of photobleaching on the measurement accuracy and error was negligible.

**Fig. S4. Performance of FFTPE.** The normalized error of OUF and measurement error of orientation varied with the number of OLID periods with SNR of 20 dB. The results changed with and without photobleaching at 0.1, 0.3, 0.5 of OUF, respectively. The mean value is the position of the marker symbol, and the bar indicates the standard deviation. pb, photobleaching.

# Supplementary Note 5: Performance of OLID-SDOM in simulation

The performance of OLID-SDOM, containing the orientation and OUF measurement precision and resolution, was simulated here. In this Supplementary Note, the simulated samples convolved with a point spread function (PSF) in 1.4 of NA and 488 nm of wavelength. Gaussian noise was added with fixed SNR and Poisson noise to generate the simulation images.

**SNR improvement by OLID:** In this simulated sample, 1300 sample points were randomly generated in the image of 512$\text{×}$512, and their OUF and orientation were also randomly generated. We only conducted OLID operation and found that SNR could be improved by 10 dB (**Table S1**).

| **Table S1. Changes of SNR before and after OLID** | |
| --- | --- |
| **SNR of acquired images** | **SNR after OLID** |
| 11.5 dB | 21.9 dB |
| 21.9 dB | 32.0 dB |
| 31.4 dB | 41.2 dB |

**Measurement Precision of Orientation and OUF:** This simulation sample was same as the sample in above part “**SNR improvement by OLID**”. The simulation images were reconstructed by OLID-SDOM to represent the resolution of OLID-SDOM. The measurement precision of OUF was the standard deviation of ((calculation OUF – preset OUF)/ preset OUF), and the measurement precision of orientation was the standard deviation of the measurement error of orientation for all points in the sample. As shown in **Fig. S5a**, the higher the SNR of the raw data was, the higher the measurement precision of OUF and orientation would be. The precision of orientation could reach 4.7$\text{°}$, and the precision of OUF could reach 0.14.

**Resolution of OLID-SDOM**: We generated a simulation of dual-point with different orientations from 0$\text{°}$ to 90$\text{°}$ to demonstrate the resolution with different OUF and SNR. As shown in **Fig. S5b**, a worse SNR would result in artifacts in the reconstructed image, but the resolution would not deteriorate. In addition, the resolution was related to both OUF and orientation difference $\text{∆α}$. In **Fig. S5c**, the lowest resolution was 200 nm (OUF=1.0, $\text{∆α}$=0$\text{°}$). The higher $\text{∆α}$ was, the higher resolution was. With the condition of 50$\text{°}$, the larger OUF was, the lower resolution was. With the condition of 50$\text{°}$, the larger OUF was, the higher resolution was. When 90$\text{°}\text{ }$ and OUF=1.0, two neighboring emitters with a spatial distance of 80 nm could be separated. Referring to the orientation mapping (**Fig. S5d**), the distance of 140 nm could be distinguished when 0$\text{°}\text{ }$ and the distance of 50 nm could be distinguished when 0$\text{°}$.

**Fig. S5. Performance of OLID-SDOM.** (a) The variation curves of the measurement precision of OUF (left) and orientation (right) under different SNR of raw data. (b) The intensity profile of reconstruction points with different SNR. Left and right panel were profiles at 1.0 of OUF, with 10$\text{°}$(left) and 90$\text{° (right) of }\text{∆α}$, and 200 nm (left) and 90 nm (right) of distances. (c) The resolution distribution of OLID image (left) and G_ac_ image (right) with OUF and $\text{∆α}$. (d) The orientation mapping in different conditions was labelled. The white arrows were the calculation orientation of the sample points. Scale bar: 100 nm.

# Supplementary Note 6: Comparison between 2D deconvolution and 3D deconvolution

We compared the point spread function (PSF) in $(x,y)$ 2D coordinate system and in $(x,y,\theta)$ 3D coordinate system. Two neighboring emitters could be used to illustrate the resolution enhancement in $(x,y,\theta)$ 3D coordinates. For simplicity, the intensity images with or without polarization were plotted in $(x,\theta)$ coordinates and were compared. When only spatial information was used, the resolution *R* was the minimum distance to resolve the neighboring spots (**Fig. S6b**). However, when the orientation difference *dθ* between the emitters was considered, the distance *R*’ contained not only a spatial distance but also a distance in *θ* axis (**Fig. S6c**). When *R* and *R*’ remained the same, the actual spatial resolution in $(x,y,\theta)$ 3D coordinate would be higher if their orientation difference was larger. Besides, we found that the cosine-square curve of polarization modulation in one period could be approximated to the Gaussian curve of the PSF with its $FWHM_{\theta}$ of 84° (**Fig. S6a**). If the *FWHM* (full width at half maximum) of the PSF was simply taken as resolution, the spatial resolution in $(x,y,\theta)$ 3D coordinate would be $\sqrt{2}$ times better when the orientation difference between the emitters was 60° (roughly $\frac{\sqrt{2}}{2}FWHM_{\theta}$ ).

**Fig. S6. The comparison between 2D deconvolution and 3D deconvolution.** (a) Gaussian fitting of the cos-square function. The cos-square function was $\text{cos}{\text{(}\text{x}\text{-90)}}^{2}$ and the fitted Gaussian function was $\text{1.03*}\text{e}^{\text{-}{\text{(}\frac{\text{x}\text{-90}}{50.65}\text{)}}^{2}}$, whose *FWHM* was calculated as 84°. The adjusted R-square of fitting was 0.9921. (b) The intensity distribution of two neighboring emitters, when polarization was not considered. (c) The intensity distribution of two neighboring emitters in (*x*,*y*,*θ*) 3D coordinate.

# Supplementary Note 7: Comparison between SDOM, SPoD and OLID-SDOM

For the imaging model in **Eq.4**, the reconstructed sample $G_{dc}$and $G_{ac}$could be estimated by solving the optimization problem using or FISTA^3^ algorithm:

|  | $\text{OLID-SDOM:}\mathop{\mathrm{argmin}}\limits_{G_{dc},G_{ac},b} \left\Vert\mu(g(R,\theta))-I_{\det}(R,\theta)\right\Vert_{2}^{2}$ | **Eq.S1** |
| --- | --- | --- |
|  | $\text{SDOM or SPoD}:\mathop{\mathrm{argmin}}\limits_{g,b} (\sum_{\theta} \sum_{R} (\mu-I_{\det}\log\mu)+\lambda_{1}\left\vert g\right\vert_{1}+\lambda_{2}\left\vert b\right\vert_{1})$ | **Eq.S2** |

Where, *g* and *b* were the reconstruction images and background, $\text{μ}\text{ }$ in **Eq.S2** was same as the $\text{μ}$ in **Eq.S1** and **Eq.4**, $\text{λ}_{1}$ and ${\text{ }\text{λ}}_{2}$ were the hyper-parameters to balance the fidelity term and sparsity term. Importantly, the previous SDOM^4^ or SPoD^5^ algorithm contained a sparsity constraint ($\lambda_{1}\left|g\right|_{1}+\lambda_{2}\left|b\right|_{1}$) of the sample. However, the sparsity of the sample $G_{dc}$and $G_{ac}$ was not imposed in the optimization equation of OLID-SDOM since $G_{ac}$ had only sparsity in the dipole orientation axes and $G_{dc}$ was not sparse at all. Spatial sparsity only existed in sparse samples such as individual images of single molecular imaging and was not true for widefield imaging, including SDOM or SPoD. Besides, the Poisson statistics in SDOM or SPoD were not used in OLID-SDOM because the lock-in process had the denoising effect itself.

Instead, the least-squared estimation was applied in SDOM. There, the dipole orientation was extracted by an equivalent transformation between $G_{dc}$and $G_{ac}$ by FFTPE (**Eq. 5-9**), which described the fact that multiple dipoles in the same position could be equivalently taken as one ensemble dipole with both polarization variant component B and polarization invariant component A (**Supplementary Note 4**). FFTPE could calculate the orientation of all the pixels **on a whole image simultaneously** and was **faster** than the least-squared fitting method.

The simulated data were the fluorescent images of two neighboring emitters with a distance of 100 nm, whose fluorescence anisotropy was 0.5. The *FWHM* of the simulated PSF function was 250 nm. As shown in **Fig. S7**, SPoD and OLID-SDOM were able to resolve these two points in reconstruction images, while SDOM couldn’t. Moreover, as can be seen from the lower panel in **Fig. S7**, OLID-SDOM has higher angular accuracy.

In conclusion, OLID-SDOM has four significant advantages, comparing with SDOM or SPoD:

1). The optical Lock-in Detection used in OLID-SDOM separated AC component and DC component and removed noise from other frequency components.

2). OLID-SDOM didn’t use the sparsity term used in SDOM and SPoD, which reduced the artifacts under low SNR.

3). The deconvolution of SDOM was only in the spatial domain, while OLID-SDOM and SPoD introduced the $\text{θ}$ information into the deconvolution process. So the 3D deconvolution of SPoD was in the spatial and polarization domains, which provided better resolution than SDOM ($\sqrt{2}$times better than the only deconvolution process of average image). Therefore, reconstructed $G_{ac}$ image could achieve a super-resolution, even in low fluorescence polarization samples.

4). SPoD did not provide any information about dipole orientation, but both SDOM and OLID-SDOM did. In addition, the speed of FFTPE is faster than fitting in SDOM.

OLID-SDOM adopted the advantage of both SDOM and SPoD, achieving the improved super resolution of $G_{ac}$and utilizing an equivalent transformation to extract dipole orientations.

**Fig. S7. Comparison of the iteration process among Deconvolution, SDOM, SPoD, and OLID-SDOM.** The upper panel is the intensity images, while the lower panel is the section in (*x*,*θ*) coordinate (for SDOM, SPoD, and OLID-SDOM).

# Supplementary Note 8: Denoising of polarization modulation data

Keller et al^6^. found that each pixel region contained an insufficient fluorescence signal from polarization modulation, compared with Poisson noise that fluctuated with the same amplitude. Though the polarization modulation data contained only one period of data, we found that after 5×5 binning of the neighborhood pixels and due to the enhanced ensemble dipoles, almost all of the spine head areas showed significant polarization modulation, clearly distinguishable from background, since the noise from the background area was more stochastic. In **Fig. S8**, the polarization modulation signal was much higher than that of the background after pixel binning denoising from the neuronal spine dataset. This clearly revealed that polarization modulation could be well separated from noise. Meanwhile, the polarization modulation also revealed the apparent change in dipole orientation.


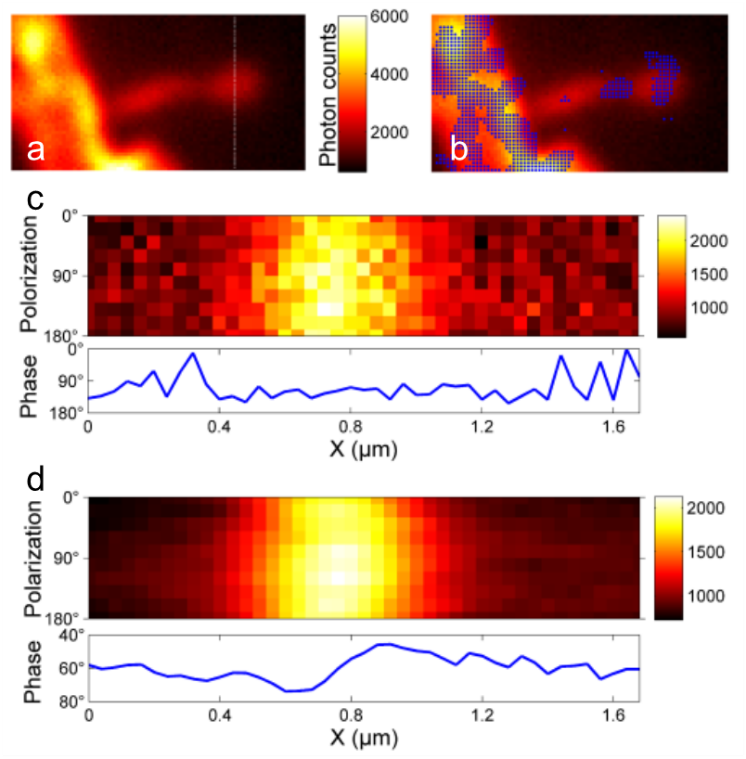


**Fig. S8.** **The performance of denoising of polarization modulation data.** (a) and (b) are the wide-field image (a) and orientation mapping (b) of the neuronal spine. (c) The upper is the photon counts within a modulation period at the vertical line in (a), while the lower is the phase calculated by demodulation. (d) is the result of binning of (c).

As high-frequency signals were removed, only the zero-frequency and modulated signals with the same frequency was retained in OLID-SDOM method, so most of the noise was removed. Compared with the average method, OLID-SDOM had better denoising capability (**Fig. S9**).

**Fig. S9. OLID-SDOM method to enhance the discernment of dipole orientation.** (a) The intensity trace of the experimental polarization modulation data of the DNA origami sample (left panel). After OLID, the sine wave of modulation was abstracted (right panel). (b) The decreasing of noise using OLID (blue), or averaging using simulated data (red).

# Supplementary Note 9: 3D Imaging with OLID-SDOM

The principle of OLID-SDOM super-resolution via polarization demodulation is suitable for three-dimensional imaging. With the z-scanning acquisition and polarization modulation applied during acquiring each frame acquirement, the 3D super-resolution intensity imaging could be achieved with the in-plane fluorescent dipole orientation measurement. Benefitted from the resolution enhancement of OLID-SDOM, the shell structure of a 1-µm diameter fluorescent bead could be clearly resolved (**Fig. S10a**). **Fig. S10c** showed the super-resolution 3D spatial distributions of the actin bundles in HeLa cell with the depth of actin color-coded. **Fig. S10b** showed three crossing actin fiber bundles, with the upper two perpendiculars to the plane and the lower one remaining in the plane, while **Fig. S10c** showed the super-resolution 3D spatial distributions of the actin bundles in HeLa cell with the depth of color-coded actin.

**Fig. S10. 3D imaging of OLID-DOM.** (a) 3D imaging results of 1 µm fluorescent beads labeled with Alexa Fluor 647 on the surface. The x-y, y-z, and x-z orthogonal projections were displayed as well as the isotropic surface of the shell. (b) 3D imaging results of Alexa Flour 488 labeled F-actin in fixed BAPE cells. The depth information was coded in pseudo-color, with the range from 0 µm to 1 µm. (c) x-z M.I.P (max intensity projection) image of the rectangle marked on (b) of super resolution results (upper panel) and wide filed results (lower panel). The three crossing actin fiber bundles marked by the dotted box corresponded to those marked by arrows in (b). (d) Intensity profile of the vertical line marked on (c), demonstrating a sub-diffractional diffraction axial resolution (red curve) of 3D OLID-SDOM. Scale bar: 1 µm.

# References

1 Edelstein, A. D. *et al.* Advanced methods of microscope control using μManager software. *J. Biol. Methods* **1** (2014).

2 Richard, B. *LabVIEW: Advanced Programming Techniques*. (CRC Press, 2006).

3 Beck, A. & Teboulle, M. A fast iterative shrinkage-thresholding algorithm for linear inverse problems. *SIAM Journal on Imaging Sciences* **2**, 183-202 (2009).

4 Zhanghao, K. *et al.* Super-resolution dipole orientation mapping via polarization demodulation. *Light: Science & Application* **5**, e16166-e16166 (2016).

5 Hafi, N. *et al.* Fluorescence nanoscopy by polarization modulation and polarization angle narrowing. *The Journal of Chemical Physics* **11**, 579-584 (2014).

6 Frahm, L. & Keller, J. Polarization modulation adds little additional information to super-resolution fluorescence microscopy. *Nature Methods* **13**, 7 (2015).
